# *AnXplore*: a comprehensive fluid-structure interaction study of 101 intracranial aneurysms

**DOI:** 10.3389/fbioe.2024.1433811

**Published:** 2024-06-24

**Authors:** Aurèle Goetz, Pablo Jeken-Rico, Ugo Pelissier, Yves Chau, Jacques Sédat, Elie Hachem

**Affiliations:** ^1^ Computing and Fluids Research Group, CEMEF, Mines Paris PSL, Sophia Antipolis, France; ^2^ Department of Neuro-Interventional and Vascular Interventional, University Hospital of Nice, Nice, France

**Keywords:** intracranial aneurysm, haemodynamics, fluid-structure interaction, open-source dataset, arterial wall tissue modelling

## Abstract

Advances in computational fluid dynamics continuously extend the comprehension of aneurysm growth and rupture, intending to assist physicians in devising effective treatment strategies. While most studies have first modelled intracranial aneurysm walls as fully rigid with a focus on understanding blood flow characteristics, some researchers further introduced Fluid-Structure Interaction (FSI) and reported notable haemodynamic alterations for a few aneurysm cases when considering wall compliance. In this work, we explore further this research direction by studying 101 intracranial sidewall aneurysms, emphasizing the differences between rigid and deformable-wall simulations. The proposed dataset along with simulation parameters are shared for the sake of reproducibility. A wide range of haemodynamic patterns has been statistically analyzed with a particular focus on the impact of the wall modelling choice. Notable deviations in flow characteristics and commonly employed risk indicators are reported, particularly with near-dome blood recirculations being significantly impacted by the pulsating dynamics of the walls. This leads to substantial fluctuations in the sac-averaged oscillatory shear index, ranging from −36% to +674% of the standard rigid-wall value. Going a step further, haemodynamics obtained when simulating a flow-diverter stent modelled in conjunction with FSI are showcased for the first time, revealing a 73% increase in systolic sac-average velocity for the compliant-wall setting compared to its rigid counterpart. This last finding demonstrates the decisive impact that FSI modelling can have in predicting treatment outcomes.

## Introduction

Intracranial aneurysms (IAs) are pathological dilations of blood vessels hosted by around 3% of the world’s population according to prevalence studies (Vlak et al., 2011; Cras et al., 2020). In most cases, they form over years without causing any symptoms to the patient. However, the weakened bulge tissue may not withstand perpetual exposure to stresses from the cardiac pulse, leading to a sudden rupture. The subsequent subarachnoid haemorrhage often causes death or permanent disabilities (Ingall et al., 2000). When unruptured IAs are identified, physicians have to decide whether to operate or not. Despite recent advancements in endovascular treatment, all clinical interventions carry a non-negligible threat (Naggara et al., 2012; Pierot et al., 2018). Consequently, it is necessary to enrich the current risk evaluation protocols, which are mainly based on the size, shape and location of the aneurysm bulge, with more personalized physics-based criteria. Research efforts are thus made to simulate patient-specific haemodynamics of IAs through Computational Fluid Dynamics (CFD) to assist physicians in clinical decision-making.

Promising results have already been reported in that regard. Firstly, several haemodynamic indicators such as the Wall Shear Stress (WSS) computed on digital replicates of patient-specific IAs have been successfully correlated with aneurysm growth (Cebral et al., 2019), providing new risk metrics. Secondly, the analysis of simulated haemodynamics has helped understand better the disease’s progression through complex remodelling pathways (Malek, 1999; Meng et al., 2014). Images of the bulge taken during surgical interventions have been correlated with CFD simulations to link local haemodynamic descriptors with the pathological tissue characteristics (Furukawa et al., 2018; Cebral et al., 2019). This could help predict the flow-driven weakening of the aneurysm dome through simulations to better forecast a potential rupture. Such research initiatives should ultimately lead to precise risk assessment tools able to find the balance between the clinical operation risk and the future threat of haemorrhage. Lastly, these simulations can aid in evaluating the outcome of prosthetic devices such as stents prior to an endovascular operation (Peach et al., 2015; Ouared et al., 2016) and recent work has shown how flow diverters can be tailored to patient-specific cases towards optimal individual treatment (Hachem et al., 2023).

The development of CFD simulations of IAs has raised multiple scientific challenges, ranging from the consistent generation of meshes for intricate vessel geometries to the resolution of coupled systems of equations involving adequate boundary conditions and multiple parameters (Janiga et al., 2015; Jeken-Rico et al., 2024). Among the remaining research goals stands the modelling of the complex interaction between the blood flow and surrounding vessels through Fluid-Structure Interaction (FSI). Haemodynamic risk metrics such as the WSS and Oscillatory Shear Index (OSI) have been shown to be mispredicted for some IAs when considering arteries as fully rigid (Torii et al., 2009; Brambila-Solórzano et al., 2023), raising concerns about the ability of sole inner haemodynamic simulations to accurately predict rupture risk. As a result, a few research teams have developed FSI in this field and modelled the hyperelastic behaviour of arterial tissue (Torii et al., 2008; Bazilevs et al., 2010; Valencia et al., 2013) with recent contributions pushing the limits of simulation spatiotemporal resolution to reveal vibrations and instabilities in IAs (Souche and Valen-Sendstad, 2022; Bruneau et al., 2023). Recently, brain aneurysm pulsations have even been directly observed through clinical imaging (Hayakawa et al., 2013; Vanrossomme et al., 2015; Stam et al., 2021; Zhou et al., 2022), providing new perspectives to the field. Whereas it has long remained impossible to image brain arterial deformations due to the scales at hand, the development of precise electrocardiography-gated 4D-CTA (Computed Tomography Angiography) is progressively changing the trend and future FSI models will strongly benefit from additional *in-vivo* data. Therefore, there is an additional urge to investigate FSI models and compare simulated haemodynamics to standard rigid-wall approach results in order to measure the sensitivity of classically employed risk assessment metrics to the type of wall modelling. Furthermore, as existing studies reported results for a few isolated aneurysm cases, the large-scale statistical analysis of the complex interplay mechanisms occurring between the blood flow and the enclosing vascular tissue is still missing.

Indeed, existing studies investigating FSI for IAs have reported results based on a few cases with maximum cohort sizes of five (Bazilevs et al., 2010; Valencia et al., 2013; Voß et al., 2016). This is easily understandable as generating computational domains and simulating FSI for numerous cases are both notably computationally and time-intensive tasks. The computational cost constraint is so significant that it has motivated recent research to use static structural analysis as a surrogate to compute solid wall stresses for rupture risk assessment, omitting haemodynamic metrics but achieving a remarkable 165-fold speedup along with relatively accurate stress predictions (Sun et al., 2022). While improving the efficiency of underlying solvers and FSI coupling schemes can save computational time, a straightforward way of alleviating the overall cost of FSI studies is to rely on synthetic data, thereby eliminating the need for medical image segmentation. Another is to use idealized geometries, limiting the complexity and variance of the problem and allowing conclusions to be drawn with smaller cohorts. This is a natural way of exploring a new research question in small but controlled steps. For rigid-wall CFD simulations, idealized IA geometries have been investigated (Hassan et al., 2005; Baharoglu M. et al., 2010; Ramalho et al., 2013), showing the sensitivity of haemodynamics towards various geometrical parameters and modelling assumptions. In this work, we introduce a new dataset that simultaneously facilitates the analysis of obtained FSI results by its semi-idealized nature and brings a consequent cohort of 101 geometries. Following our reproducibility goals for IA simulations, the entirety of the used meshes and required details to replicate the results are provided. The dataset, referred to as *AnXplore*, consists of real aneurysm shapes mounted on an idealized toroidal artery. The common arterial shape enables us to explore different bulge geometries, eliminating the variance caused by the vascular environment. Furthermore, it allows for a fully automatic treatment of boundary conditions and a consistent generation of computational domains, thus drastically cutting the pre- and post-processing time. In this work, we use the dataset to assess the sensitivity of simulated haemodynamics regarding the treatment of aneurysm walls. We explore the relevance of modelling aneurysm pulsations in different scenarios, including the case of a flow-diverter-stented aneurysm. To the best of our knowledge, it is the first time such a configuration has been simulated taking into account a complete FSI setting. After the dataset is introduced, transient haemodynamic simulations are presented for the 101 cases both using rigid and deformable arterial walls. Direct comparisons between haemodynamics resolved with the two methods are presented to highlight the relevance of FSI in IAs depending on the aneurysm bulge shape.

## Materials and methods

### AnXplore: A novel functional dataset for studying intracranial aneurysms

**FIGURE 1:**
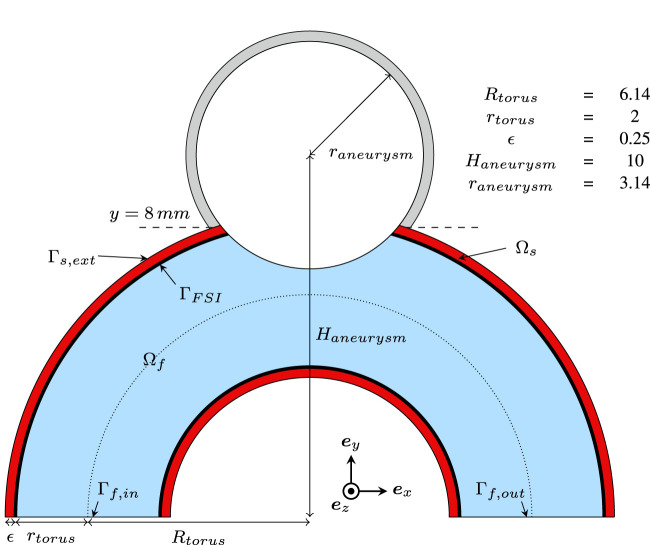
2D cut of the basic geometry used for building the dataset. Dimensions are given in mm.

**FIGURE 2:**
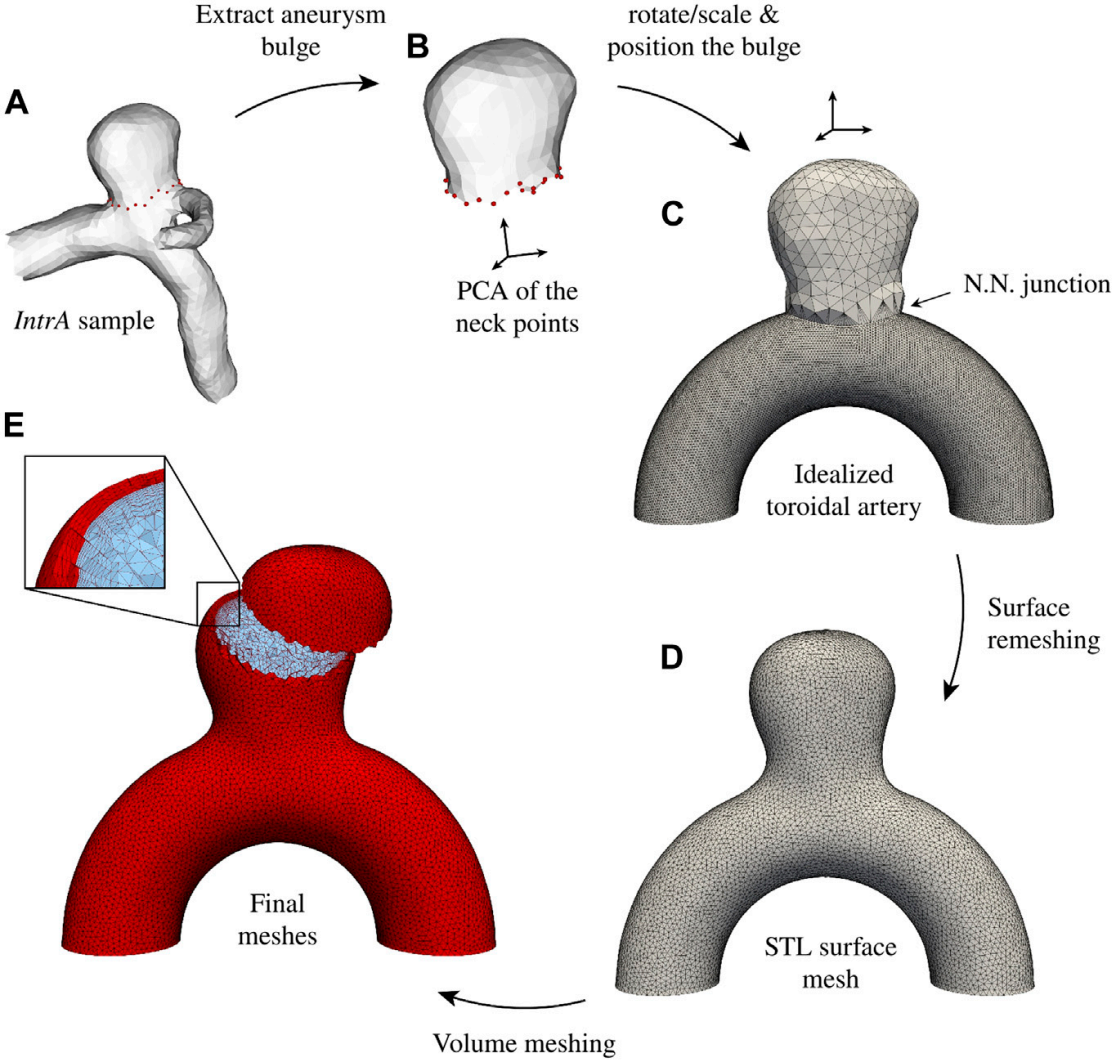
*AnXplore* generation pipeline. **(A)** The bulge is extracted from the original *IntrA* (Yang et al., 2020) mesh. **(B)** A PCA of the neck points is implemented and **(C)** the bulge is rotated/scaled to mount it on the toroidal artery. After Nearest Neighbors (N.N.) nodes are connected, the surface is remeshed **(D)** to obtain a homogeneous mesh size. **(E)** Lastly, volume meshes are generated.

The proposed dataset is inspired by our previous work (Goetz et al., 2024), which introduced an idealized aneurysm geometry for studying FSI-related phenomena. This benchmark geometry, composed of a spherical aneurysm intersecting a toroid, is displayed in Figure 1. The artery diameter is 4 mm which has been reported to be a realistic calibre for the last segments of a human Internal Carotid Artery (ICA) based on consistent measurement involving 70 patients (Baz et al., 2021). This appears to be a relevant location for a sidewall aneurysm case, as more than half of them are located around the ICA siphon or close to the ophthalmic artery branching (Baharoglu M. I. et al., 2010; Baharoglu et al., 2012). The spherical bulge has been greyed out in Figure 1 as it is meant to be replaced with realistic shapes in this work to explore a manifold of aneurysm morphologies and their impact on CFD modelling assumptions. With that aim, we employed the open-source *IntrA* dataset (Yang et al., 2020), which consists of 116 IAs reconstructed from time-of-flight angiographic scans by medical experts. Bulge shapes have been adapted from this dataset and mounted on our simplified curved artery as described in Figure 2, following the subsequent steps.

• The dome and neck points are extracted from the *IntrA* sample (see Figure 2A).
• A Principal Component Analysis (PCA) is applied on the neck points shown in red in Figure 2B.
• Using the barycenter coordinates of the neck curve, along with its PCA, the aneurysm is translated, rotated and scaled to approximately fit the neck of the idealized artery.
• This coarse fit provides a starting point for employing an iterative closest point algorithm (Arun et al., 1987) to best align the two point clouds.
• The optimally positioned dome is additionally shifted up by a small margin (0.3 mm) and upscaled by a factor 1.1, to give space for new surface triangles, which join the surface meshes together.
• Finding nearest-neighbour pairs between the two neck curves, triangles are created (see Figure 2C).
• The obtained surface is refined and optimized in alternating turns with Gmsh (Geuzaine and Remacle, 2009) and Mmg (Dobrzynski, 2012).
• Finally, the volumetric meshes are produced, which will serve as computational domains for subsequent numerical simulations. The solid is generated by the outward extrusion of the surface mesh, consisting of four layers reaching a total thickness of *ϵ* = 0.25 mm, which corresponds to commonly prescribed values (Torii et al., 2009; Bazilevs et al., 2010; Valencia et al., 2013; Cebral et al., 2014). For the fluid, after generating boundary layers, the core volume is filled with isotropic tetrahedra. As velocity gradients are computed through post-processing, mesh resolution is of major importance, especially close to the walls. Here, we employ a 1.2 geometrical progression between successive boundary layers with a minimal element thickness of 20 μm. The resulting meshes contain between 0.8 M and 1.6 M elements for the fluid domains and between 0.3 M and 0.6 M for the solids.

### Modelling haemodynamics coupled with arterial tissue deformation

All simulation results presented in this study have been obtained using our C++ in-house code, which is a highly parallelized stabilized finite-element library relying on PETSc (Balay et al., 1998) for the resolution of linear systems. This section outlines the utilized numerical framework, beginning with an overview of the fluid and solid solvers. Subsequently, the implemented FSI strong-coupling scheme and the numerical simulation protocol are detailed. The presented FSI framework has been validated on benchmarks and aneurysm test cases in a previous study (Goetz et al., 2024).

#### The ALE incompressible navier-stokes equations

This work employs an Arbitrary Lagrangian-Eulerian (ALE) (Hirt et al., 1974) description of the fluid dynamics, where Ω_*f*,*t*_ defines the fluid spatial domain at time *t* ∈ [0, *T*] and ***ψ*** is the ALE mapping from Ω_*f*,0_ to Ω_*f*,*t*_. The relative mesh velocity is denoted ***v***_***m***_. We solve the mixed formulation in velocity ***v*** and pressure *p*_*f*_ of the transient incompressible Navier-Stokes equations:

$$\nabla \cdot v=0,\;\;\rho_{f}\partial_{t}v+\rho_{f}\left(\left(v-v_{m}\right)\cdot \nabla \right)\;v-\nabla \cdot \sigma_{f}=f,\;\text{in}\;\Omega_{f,t}.$$ (1)

where *ρ*_*f*_ = 10^3^ kg/m^3^ is the fluid mass density, ***f*** the source term, and ***σ*** _***f***_ the Cauchy stress tensor. As part of our partly idealized simulation setting, blood is considered Newtonian with *μ* = 0.004 Pa s.

We employ a P1-P1 finite element discretization for solving Eq. 1, combined with a Variational Multiscale-type (VMS) method further described by Hachem et al. (2010). This approach grants accuracy and stability both regarding the inf-sup condition (Babuška, 1971) and for convection-dominated flow. In the ALE framework, the convective term in Eq. 1 is altered by the mesh velocity *v*_*m*_, which keeps coupling interfaces (Γ_*FSI*_) fitted. In this study, *v*_*m*_ is obtained by solving a diffusion equation as done in the work of Habchi et al. (2013); Breuer et al. (2012); Shamanskiy and Simeon (2021).

Once velocity and pressure are obtained, widely used IA rupture risk indicators are computed through post-processing. Of particular interest are the WSS applied by the blood flow in the bulge and the OSI. They are defined as follows.

$$\tau_{\mathrm{WSS}}=n\times \left[\left(\sigma_{f}\cdot n\right)\times n\right]=\sigma_{f}\cdot n-\left[\left(\sigma_{f}\cdot n\right)\cdot n\right]n\;\;\text{OSI}=\frac{1}{2}\left(1-\frac{\left\|\int_{t_{0}}^{t_{0}+T}\tau_{\mathrm{WSS}}\;dt\right\|}{\int_{t_{0}}^{t_{0}+T}\|\tau_{\mathrm{WSS}}\|\;dt}\right)$$ (2)

Where ***n*** is the unit normal vector at the wall, ***σ*** _***f***_ the Cauchy stress tensor, and *T* the cardiac period at hand. In this work, WSS is reported as a scalar quantity corresponding to the Euclidian norm of ***τ***_***WSS***_.

#### A hyperelastic vascular tissue

For modelling the hyperelastic nature of arterial tissue, we rely on a Neo-Hookean formulation (Rivlin, 1948), complemented with a Simo–Taylor (Simo et al., 1985) volumetric model. Following previously reported order of magnitudes (Torii et al., 2008; 2009; Bazilevs et al., 2010; Voß et al., 2016), the wall Young modulus is set to *E* = 0.75 MPa (with Poisson ratio *ν* = 0.45). This has already proved in previous work (Goetz et al., 2024) to result in pulsative aneurysm volume variations that are in line with direct *in-vivo* observations (Hayakawa et al., 2013; Vanrossomme et al., 2015; Stam et al., 2021; Zhou et al., 2022). To draw the focus solely on the bulge interaction with the blood flow, we decided to consequently increase the stiffness of the artery to *E*_*artery*_ = 10*E*. The bulge area, where the lower stiffness is prescribed, corresponds to the part of the geometry that lies above the plane *y* > 8 mm, which is indicated in Figure 1.

The compliant arterial tissue is modelled using the equations of solid dynamics in the updated Lagrangian framework denoted Ω_*s*,*t*_. We define the deformation gradient **F** and the Jacobian determinant *J* = det [**F**]. Let ***C*** denote the right Cauchy-Green strain tensor given by ***C*** = ***F*** ^*T*^ ***F***, ***S*** = *J* ***F*** ^−1^ ***σ***_***s***_ ***F*** ^−*T*^ the second Piola–Kirchhoff stress tensor and $\bar{C}=J^{-\frac{2}{3}}C$ the volumetric/deviatoric part of **C**. Using the Helmholtz formalism, the employed material’s free energy function **Ψ**(**C**) can be decomposed into its volumetric and deviatoric contributions:

$$\Psi \left(C\right)=U\left(J\right)+W\left(\bar{C}\right)=\frac{1}{4}\kappa \left(J^{2}-1\right)-\frac{1}{2}\kappa \text{ln}J+\frac{1}{2}\mu_{s}\left(\text{tr}\left[\bar{C}\right]-3\right).$$ (3)

where *κ* and *μ*_*s*_ are the bulk and shear moduli of the tissue, respectively. Building upon Eq. 3, we use the volumetric/deviatoric split of the stress and the fact that S is half the derivative of Ψ(**C**) with respect to C to yield:

$$p_{s}=2J^{-1}F\partial_{C}U\left(J\right)F^{T}=U^{\prime }\left(J\right)=\frac{1}{2}\kappa \left(J+J^{-1}\right),\;\;\text{dev}\left[\sigma_{s}\right]=2J^{-1}F\partial_{C}W\left(\bar{C}\right)F^{T}=\mu_{s}J^{-\frac{5}{3}}\text{dev}\left[{FF}^{T}\right].$$ (4)

In the near incompressible regime, the mixed formulation in displacement and pressure solved here is thus given by the following momentum and continuity equations:

$$\rho_{s}\partial_{tt}u-\nabla p_{s}-\nabla \cdot \;\text{dev}\left[\sigma_{s}\right]=0,\;\;\nabla \cdot u-\frac{1}{\kappa }p_{s}=0,\;\text{in}\;\Omega_{s,t}.$$ (5)

where $\rho_{s}J=\rho_{s_{0}}$ and $\rho_{s_{0}}=1.2\times 10^{3}\;\text{kg}/{\text{m}}^{3}$ . As for the fluid, we rely on a VMS-type method for tackling the solid finite-element problem defined by Eqs 4, 5. More details are given in (Nemer et al., 2021; Goetz et al., 2024).

#### FSI coupling

A strong FSI coupling is ensured in our partitioned approach (Felippa and Park, 1980) using an under-relaxed fixed-point algorithm. After solving the haemodynamics, fluid stresses are computed and applied as Neumann conditions on the solid coupling interface Γ_*FSI*_. Then, resolving the solid dynamics yields a displacement of the structure that is sent back to the fluid to impose the velocity as a Dirichlet boundary condition. This process accounts for one iteration of the fixed-point algorithm. This is repeated until the normed difference between two consecutive solid displacements drops below a given tolerance. Here, using an L2 norm scaled by the number of nodes in the solid mesh, the tolerance is set to 10^–5^ mm. This sub-iterating process is particularly crucial for the biological applications at hand due to the pronounced *added-mass effect* (Causin et al., 2005; Förster et al., 2007). To save some computational time and improve the method’s convergence, a momentum-accelerated Aitken Δ2 scheme is employed as described in the work of Küttler and Wall (2008); Habchi et al. (2013); Breuer et al. (2012); Eken and Sahin (2015).

#### Simulation protocol

**FIGURE 3:**
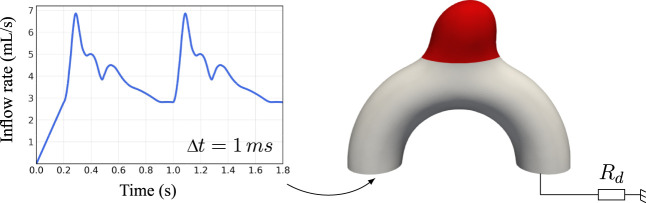
Overview of the applied boundary conditions. The inflow waveform has been adapted from Ford et al. (2005). The red region corresponds to the interchangeable bulge area changing between cases.

For each geometry of the introduced dataset, two cardiac cycles have been simulated using both a fully rigid tissue modelling approach (***u*** = 0) and a complete FSI resolution. A pulsative parabolic inlet profile is imposed on Γ_*f*,*in*_ (see Figure 1) based on the flowrate curve displayed in Figure 3. Two cardiac cycles are simulated, prefixed with a 0.2 s linear ramp for a smoother initialization. The waveform has been scaled to reach an average flow rate of 4  mL/s, which correponds to the mean ICA flow (Seymour et al., 2020). At the outlet (Γ_*f*,*out*_), a linear hydraulic resistive model *P*(*t*) = *P*_0_ + *R*_*d*_ *Q*_*out*_(*t*) accounting for the posterior vasculature is set to keep a physiological 40 mmHg pressure variation over the cardiac cycle. Given the known extreme flow rates, we employ *P*_0_ = −3.7 kPa and *R*_*d*_ = 1.31 kPa.s/mL. For the solid, nodes located on the inflow/outflow plane are kept fixed and a traction-free condition is prescribed on Γ_*s*,*ext*_. In the presented results, emphasis is drawn on time-averaged WSS (denoted TAWSS) and OSI recorded over the second cardiac cycle (from *t*_0_ = 1 s to *t*_0_ + *T* = 1.8 s) to limit transient effects related to flow initiation. Preliminary results on the benchmark spherical aneurysm geometry (Goetz et al., 2024) have shown that, while notable discrepancies are witnessed between the first and second cardiac cycles, the relative variations in TAWSS and OSI (mean, maximum, and minimum values at the aneurysm bulge) are less than 0.01% between the second and third ones, which validates this approach.

#### Flow diverter deployment and immersion

As part of this work, the effects of a flow-diverter stent on intra-saccular haemodynamics are studied in conjunction with deformable walls. The device is numerically deployed using a geometrical method similar to the one described by Bouillot et al. (2016) to account for the curvature compaction of the wires depending on the topology of the vessel. Once deployed, the obtained structure needs to be embedded into the computation mesh. Therefore, an anisotropic metric is used to refine the interfaces, as well as the boundaries of the fluid domain (Billon et al., 2017). After fitting the mesh to the wires, the nodes located in the space taken by the stent struts are removed. Boundary layers were generated to mimic the unstented cases with a progression factor of 1.2 and a minimal orthogonal mesh size of 20 μm. The resulting mesh is employed using the same simulation protocol as described above, with an additional non-slip boundary condition at the wires’ surface. No movement of the stent is modelled in response to the impinging flow.

## Results

### The *AnXplore* dataset

**FIGURE 4:**
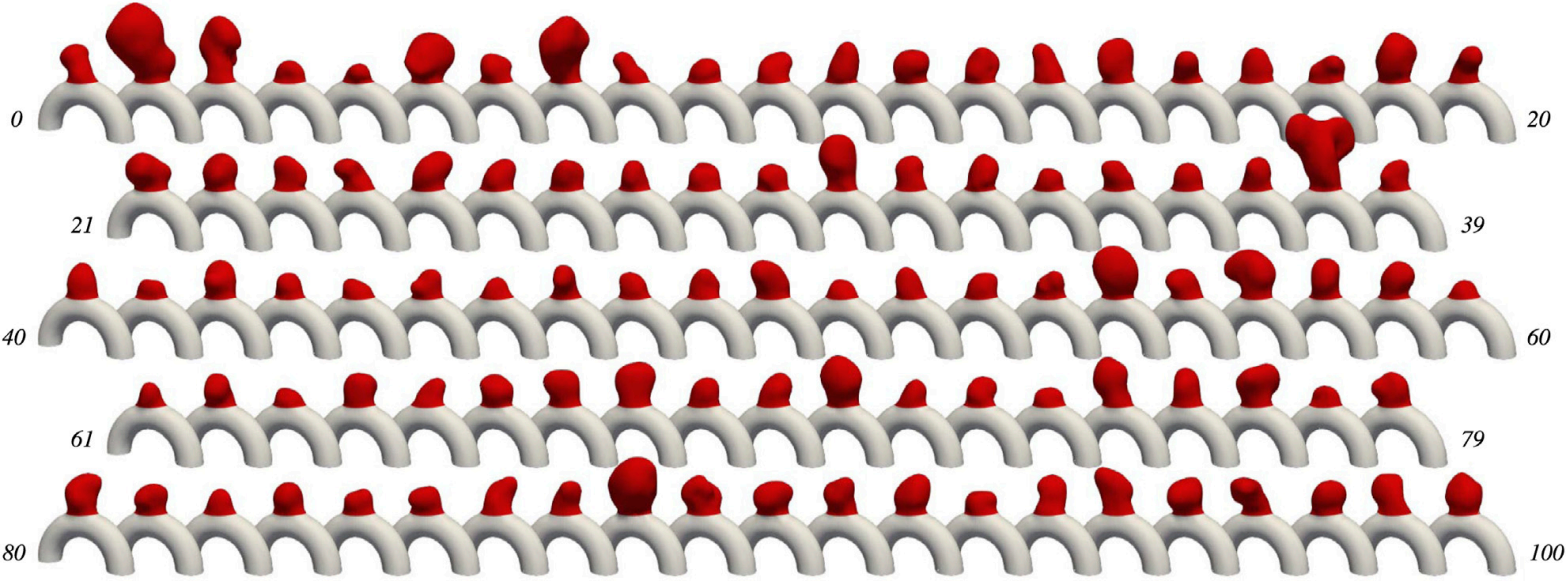
The *AnXplore* dataset. Cases are indexed from 0 to 100. The dataset is available on GitHub.

**FIGURE 5:**
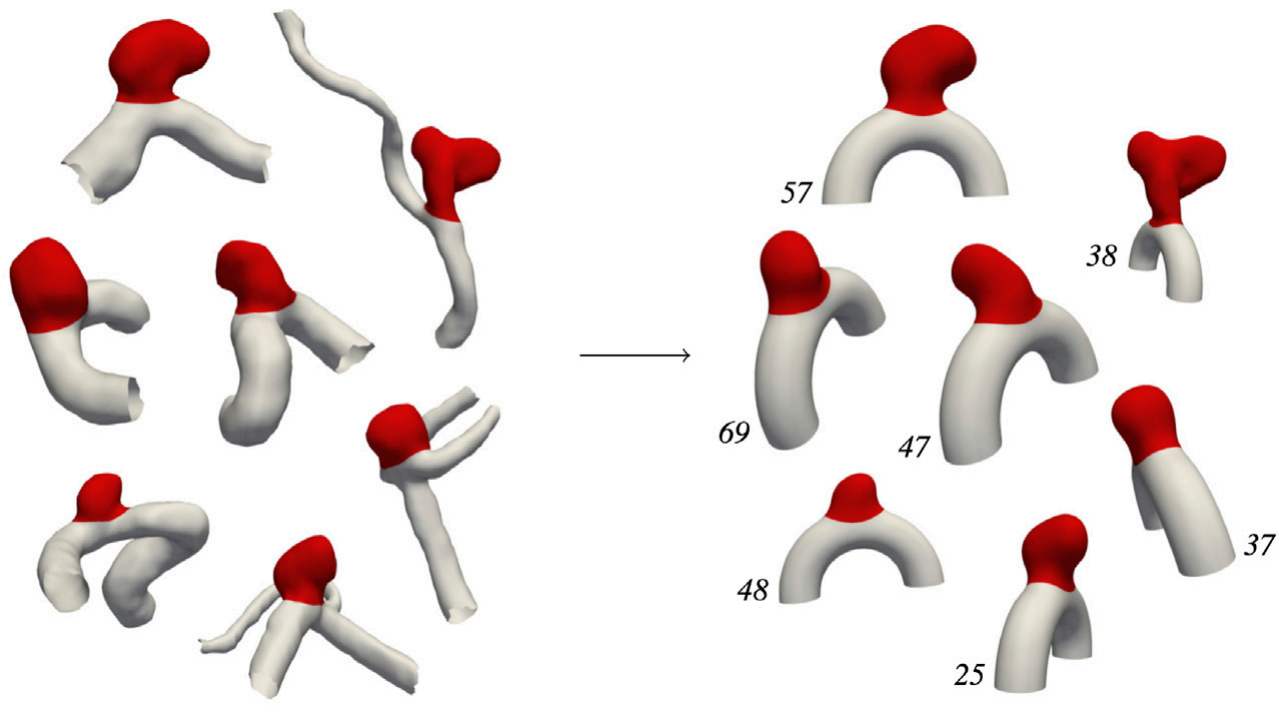
*IntrA* →  *AnXplore*. Transformation examples following the pipeline described in Figure 2.

101 aneurysm cases have been generated using patient-specific bulge geometries as described previously, yielding the *AnXplore* dataset (see Figure 4). Among the 116 original *IntrA* geometries, 15 shapes were removed either because their initial resolution was too coarse or their adaptation on our idealized artery resulted in unrealistic geometries. Some of the successful transformation examples are shown in Figure 5. The dataset, including the fluid and solid meshes of the 101 geometries, is fully available on GitHub.

**FIGURE 6:**
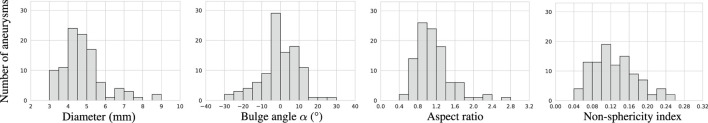
Geometrical descriptors of the investigated bulge geometries. Aneurysm diameter is computed from the bulge volume-equivalent sphere. The angle *α* is formed by the mean aneurysm dome direction with the vertical axis. The aspect ratio and Non-Sphericity Index (*NSI*) are computed as defined in the work of Dhar et al. (2008).

Histograms of geometric descriptors are given in Figure 6, highlighting the diversity of the dataset. Obtained diameters range from 3 mm to 9 mm, which is commonly considered small to medium size for IAs (Lee et al., 2015). A large span of shapes is available as reflected by the wide range of aspect ratios and non-sphericity indices (Dhar et al., 2008) that have been registered. The resulting dataset thus yields a variety of plausible aneurysm shapes. This diversity allows exploring various haemodynamic patterns occurring in sidewall aneurysms and their alteration when FSI modelling is employed. Note that cases 59, 89, and 8 correspond to the three specific shapes investigated in our previous study (Goetz et al., 2024).

### Analysis of the simulated haemodynamics using rigid arterial walls

**FIGURE 7:**
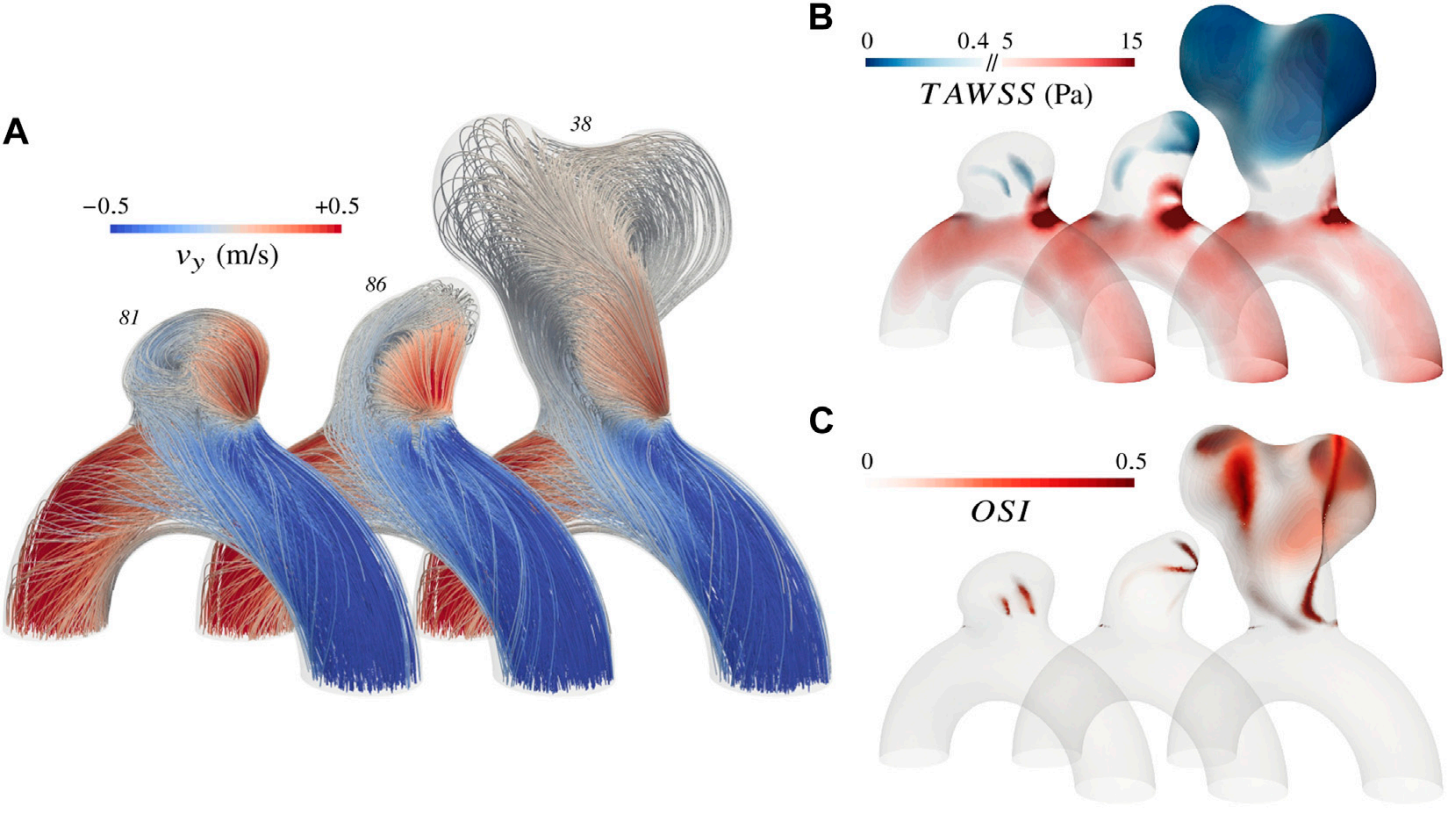
Overview of the simulated haemodynamics for three different aneurysms using rigid walls. **(A)** Systolic velocity streamlines colour-coded with the vertical component **(B)** TAWSS distribution with emphasis on extreme values as reported by Malek (1999); Meng et al. (2014) **(C)** OSI distribution.

In this section, we first introduce rigid-wall CFD simulations for the 101 cases to later use them as a reference for comparing corresponding FSI-simulated haemodynamics. For these fully rigid simulations, one case took on average 1.9 h to run on our dual processors (32-Core AMD EPYC 64-bit Processor 7,502, Advanced Micro Devices, Santa Clara, CA, United States) with 2.5 GHz base clock rate and HDR 100 interconnection. These preliminary rigid-wall results already show the range of flow patterns that can be observed in the proposed dataset. Figure 7 gives three examples of systolic haemodynamics obtained by solving the incompressible Navier-Stokes equation, along with post-processed Time-Averaged WSS (TAWSS) and OSI (see Eq. 2). Both haemodynamic metrics are associated with the well-known vascular remodelling theory (Meng et al., 2014).

It appears that the parabolic profile imposed at the inlet rapidly turns into a helical flow due to the curvature of the vessel and enters the bulge by impinging the wall close to the neck. Haemodynamics in the artery are characterized by a Womersley number of 2.2 and a Reynolds number oscillating between 220 and 520 over a cardiac cycle. The impinging jet results in larger fluid shear stresses in the neck region, both in the parent vessel and in the bulge, as reflected by the recurrent high TAWSS pattern. The slight variations of impingement intensity observed between cases are mainly driven by the angle of the bulge with the parent vessel and by the narrowness of the aneurysm. After swirling in the bulge, blood flows back into the vessel from the sides. The haemodynamic features described so far stand as the common ground of the *AnXplore* dataset. Employing a common arterial shape brings this consistency among the different cases, allowing us to highlight the sole impact of the bulge shape on the resulting intra-saccular flow. The three cases displayed in Figure 7 present a gradually increasing complexity, which reflects the general trends that have been observed in the entire dataset. Firstly, small and smoothed-shaped aneurysms, e.g. case 81, feature a steady recirculation that occupies the entire bulge without substantial changes between diastole and systole. The flow rotation usually results in small patches of high OSI, located at the center of the vortex. Secondly, aneurysms featuring a wider angle relative to the parent vessel or a higher aspect ratio commonly exhibit more complex patterns with the main swirl detaching from the wall at systole, leaving room for secondary recirculations. These cases also tend to show more pronounced variations in flow behaviour between diastole and systole. While systolic flows detach and form complex patterns, diastolic ones typically maintain adherence to the wall. This leads to flow reversal in these regions close to the dome, causing high OSI patches as for case 86. Lastly, large aneurysms generally feature hardly predictable haemodynamics due to their intricate morphology. Small disturbances of the inlet velocity propagate, causing magnified intra-saccular haemodynamic differences, and result in more pronounced OSI values over the dome (see case 38). The span of exposed flow patterns reveals the potential of the introduced dataset to cover multiple haemodynamic phenomena observed in sidewall aneurysms.

### From rigid to deformable walls: the impact on intra-saccular haemodynamics

**FIGURE 8:**
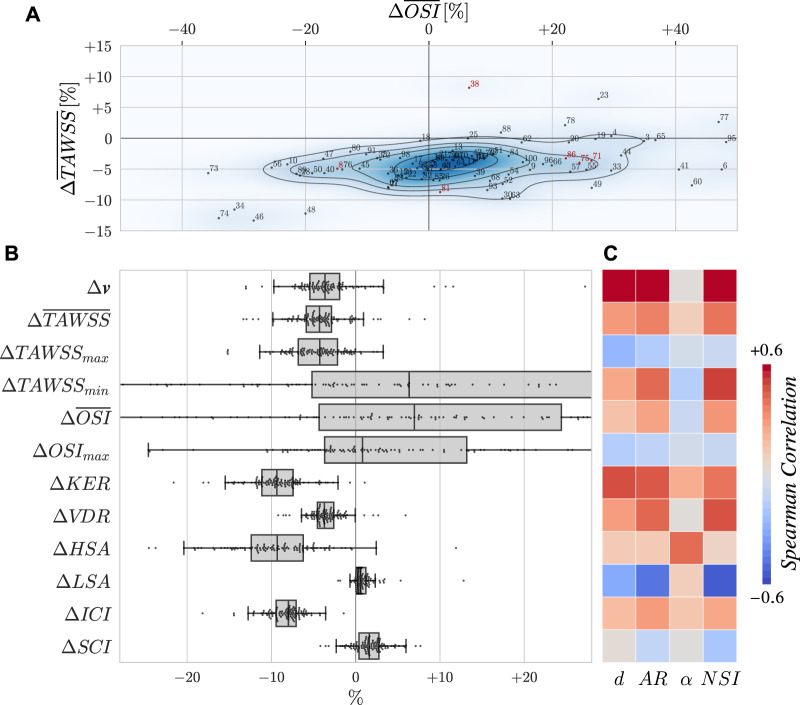
**(A)** Evolution of the surface-averaged TAWSS and OSI plotted as a density map. Indices are displayed, with red ones corresponding to cases detailed in other figures. Note that the scope of the plot excludes some points for the sake of readability. **(B)** Evolution of several haemodynamic metrics. Variations are expressed as relative changes as defined by Eq. 6. Overlined indicators are spatially averaged over the bulge surface. The velocity ***v*** is averaged in space over the bulge volume and in time over the second cardiac cycle. The remaining quantities of interest are the *KER*: Kinetic Energy Ratio, *VDR*: Viscous Dissipation Ratio, *(H/L)SA*: High/Low Shear Area, *ICI*: Inflow Concentration Index, and *SCI*: Stress Concentration Index. These indicators are also averaged in time. Definitions for them can be found in Mut et al. (2011). **(C)** Spearman correlation matrix of the flow alteration with geometrical descriptors of the aneurysms as defined in Figure 6.

**FIGURE 9:**
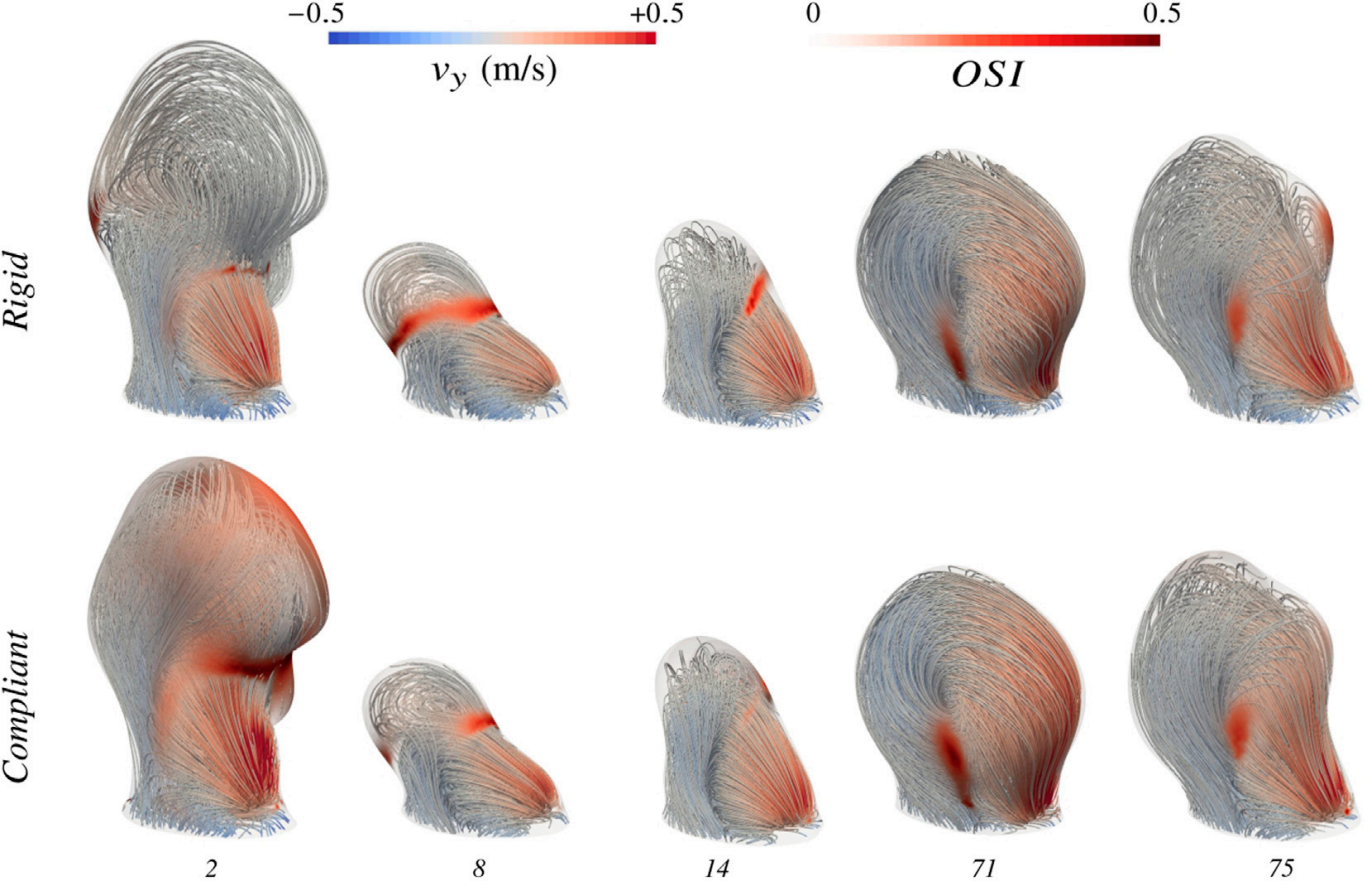
Evolution of haemodynamics moving from rigid-wall modelling to complete FSI simulations. Velocity streamlines are displayed both using rigid-wall CFD and the proposed FSI framework. High OSI regions are flagged at the surface of the bulge. Case indices are provided in the bottom row.

Strongly-coupled FSI simulations have been performed for the 101 cases by coupling the Navier-Stokes equations with our Neo-Hookean solid solver. Apart from adding tissue compliance, all simulation settings remain unchanged. Computation time represented 7.5 times the budget of rigid-wall simulations. Before inspecting in detail the haemodynamics of some notable aneurysms in Figure 9, we rely on commonly employed flow indicators such as the ones presented in Figure 8 to analyze the whole dataset’s results. These scalar quantities are easily tractable to compare FSI simulations with their rigid-wall counterparts and have been extensively correlated with aneurysm growth and rupture (Mut et al., 2011; Cebral et al., 2014; Chung et al., 2018). Each haemodynamic indicator *IND* is used to compute a relative variation between the rigid and compliant versions of the case as follows:

$$\Delta \;IND\left[\%\right]=100\times \frac{IND_{\mathrm{compliant}}-IND_{\mathrm{rigid}}}{IND_{\mathrm{rigid}}}$$ (6)

As WSS and OSI stand as the two most studied haemodynamic indicators in the CFD community, we dedicate a separate plot to show their joint evolution for the entire dataset in Figure 8A. There, a consistent drop of $\bar{TAWSS}$ can be observed when FSI is employed. While this drop has already been reported in previous studies (Torii et al., 2008; Bazilevs et al., 2010), the behaviour of oscillatory shear has on the contrary received less attention (Brambila-Solórzano et al., 2023). In this work, we have witnessed a very scattered evolution of $\bar{OSI}$ values in the bulge when wall movements are modelled. On average for the dataset, $\bar{OSI}$ increases by 20% and the standard deviation of the change reaches 74%, reflecting the large impact variability of considering tissue compliance, depending on the case geometry. The maximum change goes up to more than 600% for case 2.

Looking at the other indicators in Figure 8B, it appears that considering wall elasticity also slightly reduces velocity values in the aneurysm, hence dropping *KER* and *VDR*. Indeed, as the bulge opens due to blood pressure, neck inflow gets less concentrated (see *ICI*) and the impingement jet is smeared out, explaining the drop of high TAWSS values (*HSA*). The range of reached WSS values is thus shrunk as already reported by Goetz et al. (2024). However, stress concentration (*SCI*) does not vary substantially here, certainly due to the sidewall nature of the case that does not result in incisive flow impingement. In addition to the change in velocity, the maximum swirl intensity also occurs earlier in the cardiac cycle (6 ms earlier than the rigid counterpart on average), due to the flow-sucking effect of the inflating bulge. Aneurysm systolic volume changes of 11.9% ± 2.5% have been measured for the dataset, which is in line with literature (Vanrossomme et al., 2015). For all of the indicators reported in Figure 8B, paired Wilcoxon signed rank tests have been carried out between the rigid and deformable populations and significance (*p* < 0.05) was obtained for all of them except *OSI*_max_. As these indicators depend on the definition used for delimitating the bulge, the tests have been repeated by varying the cut height by ± 0.1 mm (that is 7.9 and 8.1 mm) and none of the presented conclusions was altered.

In Figure 8C, we provide some insights on the bulge shapes that are mostly affected by the kind of wall modelling, using geometrical descriptors. Such descriptors have been proposed with the aim of identifying straightforward rupture risk estimators (Raghavan et al., 2005; Dhar et al., 2008). Regarding $\bar{OSI}$ , it appears that elongated and irregular bulges tend to face a larger increase under consideration of wall compliance even though the correlation remains weak. The first two lines of the matrix also show that large and tall aneurysms see their intra-saccular velocity and $\bar{TAWSS}$ less reduced by compliant tissue modelling, as wall pulsations help stir the stagnant blood close to the aneurysm dome. However, even though many of the displayed trends could be commented on here, all correlation factors remain below 0.65 in absolute value. This reaffirms the intricacy of predicting the impact of arterial movements on hemodynamics based solely on bulge geometrical characteristics.

To enrich the description of haemodynamic changes beyond numeric indicators, systolic velocity streamlines with overlaid OSI are reported in Figure 9 for five of the investigated cases. This figure illustrates a diversity of responses depending on the bulge shape. While only a limited number of cases can be described in detail here, the reported mechanisms of haemodynamic change between the two kinds of wall treatment are the most notable and present in the *AnXplore* dataset. In round-shaped aneurysms like 71 or 81 (introduced before in Figure 7), the opening of the neck allows more blood to enter the bulge and displaces the vortex towards the fundus. The smeared-out recirculation carries more blood but features lower velocities as reported in Figure 8B. The opening of the bulge results in a shift of the impingement high-WSS area along with a lowering of the maximum values. As the swirl occurs deeper inside the bulge, the OSI pattern is usually shifted upwards.

To observe more pronounced effects of wall movements on resulting hemodynamics, slower flow patterns are required. In cases 8 and 14, the wide angle of the bulge with the parent vessel and the large aspect ratio induce a slow blood recirculation at the dome, which is easily perturbed by the bulge pulsations. Systolic flow variations caused by the wall movements can either develop shear oscillations over the cardiac cycle (*cf.* case 14) or realign the flow as for case 8. The distinct impact of considering tissue compliance on these two examples despite their geometrical similarity shows the complexity of the captured dynamics and the challenge posed by the pre-identification of aneurysm phenotypes mostly impacted by the kind of modelling. Evolution in case 75 is very similar to case 8. The only main difference lies in its blood recirculation being caused by the local curvature of the solid tissue at the front, which forms a little bulging where blood swirls clockwise at systole. This clockwise recirculation does not occur under diastolic conditions, which leads to the large OSI patch in the rigid configuration that disappears almost completely when wall deformations are considered. This bulging shape is indeed partly smoothed by the dome expansion and the small recirculation is reintegrated in the main swirl thanks to the wall’s inertia.

As already mentioned before, large aneurysms are the most sensitive to subtle flow variations. Compliant wall modelling preserves this tendency, as large bulges have been observed to result in the biggest $\bar{OSI}$ perturbations, usually above 50%. Case 88 (see Figure 4) stands as the only exception probably due to its regular round shape. For the other relatively large aneurysms, nearly stagnant blood flow at the dome is consequently altered when considering arterial tissue deformations. For case 2 shown in Figure 9, the jet simulated in the rigid configuration enters the bulge and hits the wall visible on the left. Then, blood slowly recirculates at the dome and exits at the back. However, haemodynamics completely change when FSI is considered, with increased OSI values at the front and a systolic velocity pattern that follows the wall movement next to the dome, leading to local flow reversal. This results in large OSI patches, which are omitted by the rigid-wall approach as reflected by the 674% increase in $\bar{OSI}$ for this case that stands as the biggest observed among the dataset. If nearly stagnant blood is consequently disturbed by aneurysm pulsations, we think it is of utmost interest to investigate the haemodynamics of aneurysms treated with flow diverters using FSI. In the following section, we study such a configuration for the first time in the context of IAs.

### Towards FSI simulations of aneurysms treated with a flow-diverter stent

**FIGURE 10:**
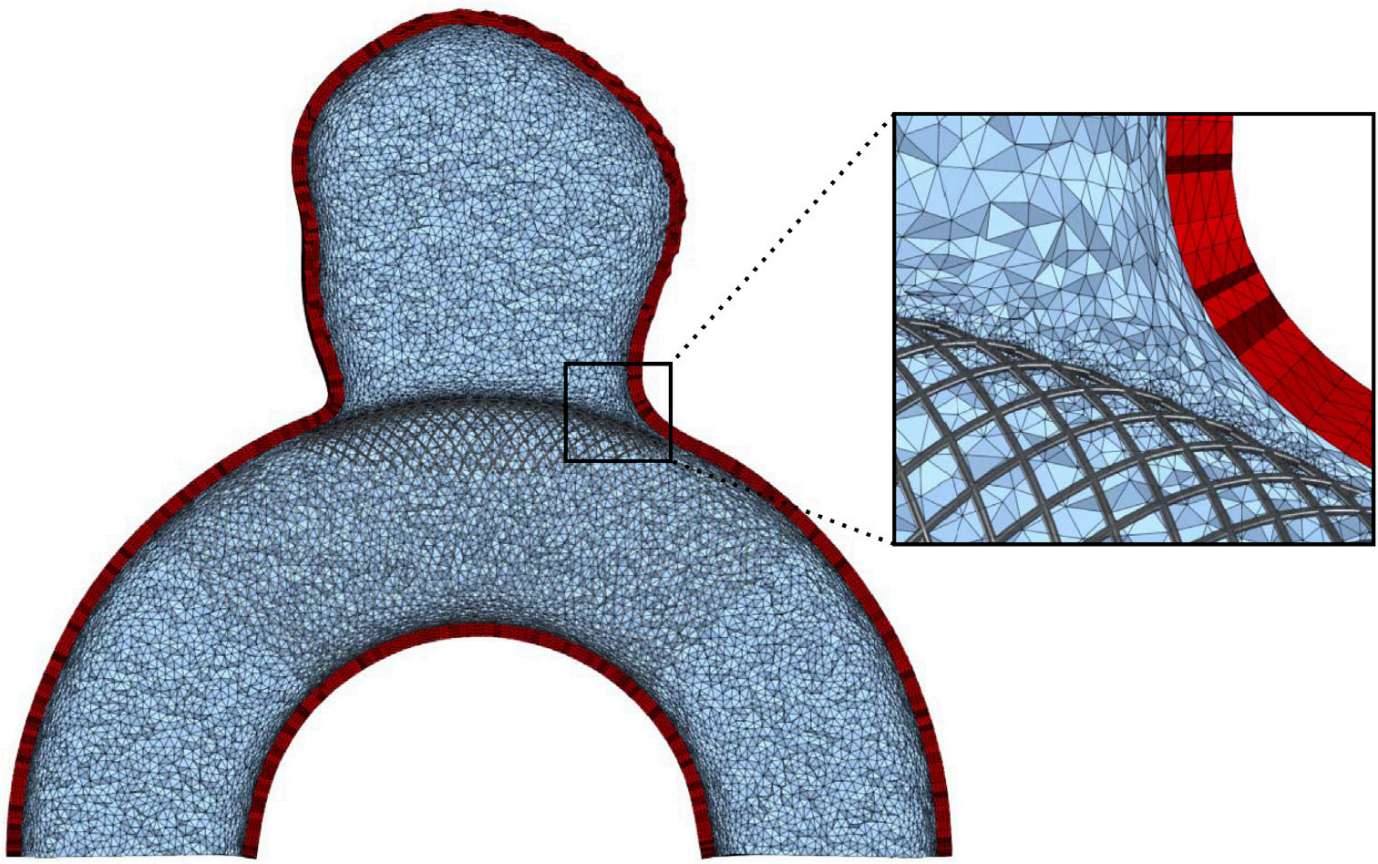
View of the mesh for case 71 with an implanted flow diverter. Low-opacity wires are not considered in the simulations. A zoom on the stent is displayed on the right. The mesh refinement at the wires goes down to h =15 μm.

The ultimate goal of flow-diverter stents is to drastically reduce the intra-saccular flow to promote thrombus formation. Substantial efforts have been made to simulate the haemodynamics of aneurysms treated with such devices (Ouared et al., 2016; Zhang et al., 2016; Sindeev et al., 2018; Sarrami-Foroushani et al., 2021) but never taking into account arterial tissue compliance. In Figure 10, we show the employed mesh of case 71 treated with a 4 mm Pipeline Embolization Device (PED; Medtronic, Dublin, Ireland) (Fischer et al., 2011). This aneurysm case has been chosen for its medium size and impingement angle, along with its regular unstented hemodynamic conditions, making it a representative example of the dataset. The stent is composed of a total of 48 braided wires of thickness 40 μm (Bouillot et al., 2014). Porosities range from 67% to 71% in front of the aneurysm neck, which is in agreement with experimentally measured values (Shapiro et al., 2014). The flow-diverter stent has been numerically deployed and immersed into the fluid mesh as described in the Methods section. The resulting fluid grid features three times more elements than the unstented one, with 3.6 M tetrahedra. Note that only the part covering the neck highlighted in Figure 10 has been included in the simulation because the rest is of minimal importance to assess flow diversion effects.

**FIGURE 11:**
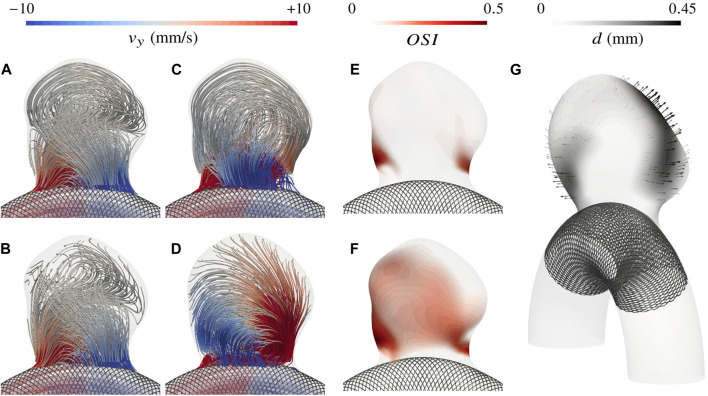
Intra-saccular haemodynamics for case 71 treated with a flow diverter. Velocity streamlines are displayed at diastole (*t*_6_=1.8 s, **(A)** rigid, **(B)** compliant) and systole (*t*_2_=1.09 s, **(C)** rigid, **(D)** compliant), revealing two different flow patterns. Then, the change in OSI between the rigid **(E)** and deformable-wall **(F)** configurations is displayed. Lastly, **(G)** shows the systolic displacement of the fluid-solid interface.

**FIGURE 12:**
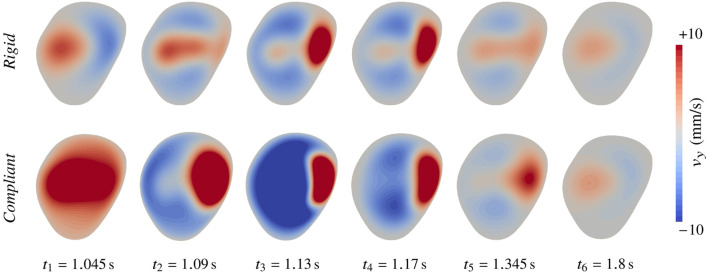
Haemodynamic alteration in case 71 treated with a flow diverter when moving from rigid to compliant wall modelling. The upward velocity component is shown at the slice defined by *y* =10 mm. *t*_2_ corresponds to peak systole.

In Figure 11, we show the simulated haemodynamics using the implanted flow diverter. Apart from the presence of the stent, the simulation settings are the same as for the previous cases. Please note that the unstented haemodynamics were already displayed in Figure 9 for this case. When a flow diverter is added, two different flow patterns are observed at diastole and systole. If blood is sufficiently slow, streamlines follow the wall and blood enters the bulge in the proximal part of the stent (see Figures 11A,B). At systole, blood accelerates and the jet enters distally, similar to the unstented configuration, causing a large recirculation in the bulge (see Figure 11D). Interestingly, this vortex happens both earlier and stronger in the deformable setting with a maximal sac-averaged velocity of 12.6 mm/s. At systolic time in the rigid configuration (see Figure 11C), the flow is still transitioning towards the swirl pattern, which fully establishes 40 ms later, peaking at 7.2 mm/s sac-averaged velocity. The maximum velocity in the bulge thus increases by 73% when tissue compliance is modelled. To better visualize the change of haemodynamic pattern, we display in Figure 12 the upward velocity component at the slice defined by *y* = 10 mm, 2 mm above the aneurysm neck. In the rigid configuration, the inflow jet continuously migrates from left to right between *t*_1_ and *t*_4_, illustrating the shift between the two aforementioned haemodynamic patterns. The flow goes back to the diastolic configuration from *t*_4_ to *t*_6_. When tissue compliance is considered, haemodynamics are influenced by the wall movements, especially when the bulge inflates (*t*_1_) and deflates (*t*_3_) following the displacement field displayed in Figure 11G. Contrary to most unstented cases, global velocity levels increase here thanks to the bulge’s suction effect. Spatio-temporal velocity in the aneurysm rises from 3.6 mm/s to 4.3 mm/s over the second cardiac cycle, which amounts to a 20% relative increase. This corresponds to velocity reductions of 91.6% and 93.1%, for the rigid and compliant configurations, respectively, when compared to their unstented counterparts. With this consequent alteration of the velocity patterns in the stented case, $\bar{OSI}$ is in turn almost doubled from 0.069 to 0.122 as reflected by Figure 11F.

## Discussion

### How important is tissue compliance in the context of intracranial aneurysms?

Numerical simulations of IAs have been developed with the aim of getting a better grasp on the disease’s progression. Metrics such as the WSS and OSI have been investigated in numerous studies for correlating them with aneurysm growth (Metaxa et al., 2010; Mut et al., 2011; Chung et al., 2018; Furukawa et al., 2018; Cebral et al., 2019) and research effort is still being pursued to refine and elaborate effective predictors (Mazzi et al., 2020; Morbiducci et al., 2020). Therefore, we decided to focus on such indicators to discuss the relevance of FSI. Aneurysmal remodelling has been shown to occur under abnormally high WSS and in blood stagnation areas through inflammation of the tissue. In these stagnation zones, the risk of thrombus or atherosclerosis also increases. Interestingly, rupture almost always occurs in such regions close to the dome, where tissue has been shown to undergo lighter structural stresses compared to highly curved neck areas (Oliveira et al., 2023). This leads to the assumption that these patches suffer from a strong tissue weakening, which is certainly the result of abnormal and oscillatory haemodynamics. Overall, risk scores like WSS and OSI embrace the known remodelling pathways as defined in the work of Meng et al. (2014). In this work, both metric’s alterations with the type of wall modelling have been showcased. High WSS values have been shown to almost consistently decrease when moving from rigid to deformable walls. The drop is intuitive as compliant vessels expand accommodating fluid stresses. The consistency is explained in the proposed dataset by the common shape of the aneurysm neck, which is the one undergoing the impinging jet. In more general circumstances using different vascular environments, WSS variations would surely cover a wider range. Moreover, simulating geometries with several outlets would further disturb the blood stream in an FSI setting as vessels with different calibres expand proportionally to their diameter under inner pressure, thus modifying the ratio of their hydraulic resistances. As opposed to high WSS, low shear stress values have been shown to increase, shrinking the range of observed values. If WSS alteration has been very consistent when taking into account tissue hyperelasticity, it is not the case for OSI. There, more significant changes have been observed, with both increasing and decreasing scores and elongated irregular aneurysms have been reported to be particularly affected. Indeed, OSI changes reflect the alteration of flow directions within a cardiac cycle and intricate aneurysm bulges often feature nearly stagnant blood regions, whose dynamics can easily be dictated by wall pulsations. However, we have shown that these OSI variations are very difficult to forecast and are tightly bound to the geometry of the case at hand.

To contextualize the significance of the documented haemodynamic alterations, it is necessary to juxtapose them with the array of uncertainties inherent to a full IA simulation pipeline. Quantifying the response variability of several CFD methods for simulating the same aneurysm case has been the topic of many studies, starting with the inspiring work of Valen-Sendstad et al. (2018), in which a considerable variability in sac-averaged WSS up to 56% has been reported between 26 participating teams. Later, challenges tried focusing on specific aspects of the pipeline, underscoring for instance the significant impact of geometry segmentation processes (Berg et al., 2018; Paritala et al., 2023). Boundary conditions have also been pointed out as crucial aspects of the simulation setting (Jeken-Rico et al., 2024; Nozaleda et al., 2024). Indeed, 25% inflow rate variations have been reported to cause TAWSS variations in the range of 30% (Øyvind et al., 2013; Nozaleda et al., 2024). In addition, concerns regarding the Newtonian assumption for blood have been raised (Fisher and Rossmann, 2009; Mahrous et al., 2020; Oliveira et al., 2021), suggesting an overestimation of WSS and underestimation of OSI in the aneurysm bulge compared to more elaborate Non-Newtonian models. However, Khan et al. (2016) prioritize segmentation and boundary conditions over the Newtonian assumption, which they deem less important. Our study contributes to this discourse by suggesting that while compliant arterial modelling does exert a clear effect, its impact may be secondary to other factors. Nonetheless, the observed variations particularly for OSI, underscore the non-negligible influence of hyperelastic arterial modelling on IA hemodynamics in certain cases. Once patient-specific boundary conditions can be obtained in clinical routine along with high-quality scans for segmentation, the fidelity of numerical simulations should be refined by taking into account the interplay between the blood stream and surrounding arterial walls. Furthermore, tissue compliance has been demonstrated to exacerbate its influence in simulations involving flow-diverter stents, which are of current interest given the growing adoption of these devices in clinical practice.

### Implications regarding numerical stent and drug outcome evaluation

Flow diverters’ main objective is reducing the intra-aneurysmal flow to the point where a thrombus forms and fills the cavity. In subsequent steps, endothelial cells populate the stent surface, ultimately preventing further growth and potential rupture (Bisighini et al., 2023). In our case, we have observed a pronounced drop in flow magnitudes inside the bulge both due to an optimal coverage of the stent and the alignment of the wires with the flow. In a previous study of a similar sidewall aneurysm, velocity reductions of more than 95% have been recorded when simulating a 80%-porosity flow diverter (Zhang et al., 2016), agreeing with our results. While the assessment of flow diverters through their flow reduction is a first helpful step, some work has taken a further step in analyzing blood residence time (Rayz et al., 2010; Menon et al., 2023). High values of the latter at near-wall regions facilitate the aggregation of platelets and the thrombus initiation. Since both the near-wall phenomena and slow recirculations have been especially sensitive to the use of non-rigid walls, we have reasons to believe that FSI simulations could have a major impact in the evaluation of endoluminal devices such as, but not limited to, flow diverters. Here, a more accurate interaction between the parent artery haemodynamics, the prosthesis, and the aneurysm dynamics could help understand why some operations have an increased risk of failure (Cebral et al., 2011). Similarly, endoluminal drug-delivery devices could benefit from increased fidelity in the wall’s vicinity, as discharge rates and drug exposure are inherently linked to near-wall haemodynamics. To the current day, many studies model drug transport mostly through steady-state rigid-wall simulations (Htay and Liu, 2005; Bozsak et al., 2014; Vijayaratnam et al., 2018), leaving considerable room for improvement in the context of the brain arteries.

### Limitations and perspectives

Although the proposed dataset is the largest that has been studied considering arterial hyperelasticity so far, the geometries investigated in this work remain partly idealized. We aimed to find a balance between simplicity and completeness to provide cases that are both easy to reproduce and resemble real aneurysm physics. The resulting dataset structure is easily set up but omits the variance induced by the vascular environment, which could be investigated as an extension of this study in future work by varying the parent vessel shape. Although this idealized setting has led to diverse plausible haemodynamic patterns and allowed drawing conclusions on the relevance of arterial compliance modelling, a gap with clinical applications remains. Essentially, having both a symmetric parent vessel and inlet profile restrains the complexity of developing aneurysmal flow when compared to the intricate nature of real tortuous arteries. Furthermore, arterial behaviour could be refined both by pre-loading the vessel structure (Bazilevs et al., 2010; Bols et al., 2013) and by employing more sophisticated constitutive modelling, such as the HGO model (Holzapfel et al., 2000) or a multi-layered hyperelastic wall treatment as presented recently by Fan et al. (2024). However, the lack of patient-specific data and general guidelines regarding pathological vascular tissue modelling impedes the meaningful prescription of precise wall properties, motivating the use of a Neo-Hookean model in our idealized setting. Progress in medical imaging will give more insights into these missing parameters, allowing precise simulation frameworks to prove their efficiency. The *in-vivo* assessment of locally varying tissue properties has drastically pushed the simulations of aortic aneurysms (Finol et al., 2013) and will surely benefit the field of IAs. Future work should focus on the indirect measurement of these wall properties through the coupling of FSI simulations with long-term tissue remodelling predictions. While these aneurysm characteristics are missing to assess solid stresses and predict rupture from a structural point of view, haemodynamic indicators can still be employed to model multiple phenomena associated with vascular diseases. As stated before, the numerical assessment of flow-diverter-induced thrombus or the evaluation of tissue exposure to diverse drugs are mainly fluid problems, which would largely benefit from a complete FSI framework without the need for exact tissue properties. The *AnXplore* dataset will be used in future work to further explore these two research directions. All in all, the results presented in this study have emphasized the impact of arterial tissue compliance on haemodynamic risk indicators depending on the bulge shape. Regions of slow recirculation have been shown to be particularly sensitive to the type of wall modelling. This has been further demonstrated in a case treated with a flow diverter, where the magnified effects of bulge pulsations have been reported for the first time. It has been shown that predicting the impact of wall motion on intra-saccular haemodynamics is not straightforward and highly case-dependent, with a share of the investigated cases being substantially altered. To reach high-fidelity simulations, this non-negligible share deserves attention and future research should consider modelling tissue hyperelasticity, especially when dealing with haemodynamic metrics computed in slow oscillatory flow.

## Data Availability

The datasets presented in this study can be found in online repositories. The names of the repository/repositories and accession number(s) can be found below: https://github.com/aurelegoetz/AnXplore.
